# A Network-Based Approach to Explore the Mechanisms of *Uncaria* Alkaloids in Treating Hypertension and Alleviating Alzheimer’s Disease

**DOI:** 10.3390/ijms21051766

**Published:** 2020-03-04

**Authors:** Wenyong Wu, Zijia Zhang, Feifei Li, Yanping Deng, Min Lei, Huali Long, Jinjun Hou, Wanying Wu

**Affiliations:** 1 Center for Modernization of Traditional Chinese Medicine, Shanghai Institute of Materia Medica, Chinese Academy of Sciences, 501 Haike Road, Pudong New District, Shanghai 201203, China; 2017e8012363117@simm.ac.cn (W.W.); zijiazhang@simm.ac.cn (Z.Z.); s19-lifeifei@simm.ac.cn (F.L.); shimbiro@163.com (Y.D.); mlei@simm.ac.cn (M.L.); longhuali@simm.ac.cn (H.L.); 2 University of Chinese Academy of Sciences, No. 19A Yuquan Road, Beijing 100049, China

**Keywords:** *Uncaria* alkaloids, network pharmacology, hypertension, Alzheimer’s disease, butyrylcholinesterase, molecular docking

## Abstract

*Uncaria* alkaloids are the major bioactive chemicals found in the *Uncaria* genus, which have a long history of clinical application in treating cardiovascular and mental diseases in traditional Chinese medicine (TCM). However, there are gaps in understanding the multiple targets, pathways, and biological activities of *Uncaria* alkaloids. By constructing the interactions among drug-targets-diseases, network pharmacology provides a systemic methodology and a novel perspective to present the intricate connections among drugs, potential targets, and related pathways. It is a valuable tool for studying TCM drugs with multiple indications, and how these multi-indication drugs are affected by complex interactions in the biological system. To better understand the mechanisms and targets of *Uncaria* alkaloids, we built an integrated analytical platform based on network pharmacology, including target prediction, protein–protein interaction (PPI) network, topology analysis, gene enrichment analysis, and molecular docking. Using this platform, we revealed the underlying mechanisms of *Uncaria* alkaloids’ anti-hypertensive effects and explored the possible application of *Uncaria* alkaloids in preventing Alzheimer’s disease. These results were further evaluated and refined using biological experiments. Our study provides a novel strategy for understanding the holistic pharmacology of TCM, as well as for exploring the multi-indication properties of TCM beyond its traditional applications.

## 1. Introduction

The *Uncaria* stem with hooks (as known as gou-teng in Chinese) is widely used clinically as a formulae medicine with anti-hypertensive effects in traditional Chinese medicine (TCM) [1,2,3]. As the main bioactive compounds in the *Uncaria* plants, *Uncaria* alkaloids have shown significant hypotensive potency in the spontaneously hypertensive rat model [1,4]. Besides the effects on the cardiovascular system, *Uncaria* plants are also documented in ancient TCM formulae that alleviate symptoms associated with the central nervous system (CNS), such as febrile seizures, dizziness, and migraines [1,2,3]. A few recent studies show that rhyncophylline, one of the most widely studied indole alkaloids from the *Uncaria* plants, has effects in reducing aggregation and deposition of amyloid proteins and inhibiting extrasynaptic *N*-methyl-d-aspartate (NMDA) receptor activation [1,2,5,6,7,8,9,10]. These studies indicate that the bioactive molecules extracted from the *Uncaria* plants may have potential in treating neurodegenerative diseases like Alzheimer’s disease (AD).

Currently, there is no cure available in reversing AD disease progression, and most approved drugs for AD treatment are for symptom alleviation, e.g., improving mental functions, reducing behavioral problems from AD, or slowing down memory decline [11,12,13]. Much effort has been made to search for the genetic and environmental factors contributing to the pathogenesis of AD among the aging population. Vascular damage, including capillary rarefaction, blood–brain barrier disruption, and consequential neuroinflammation, impairs neurovascular-coupling responses and promotes the genesis of cerebral microhemorrhages [14,15]. It explains many of the biological processes and pathogenesis of AD and this is supported by many epidemiological and experimental studies [14,16,17,18,19,20,21,22].

Impaired CNS vasculature causes permanent damage to hippocampal formation and cognitive functions [23]. It indicates that drugs used in hypertension treatment may have additional benefits in slowing down AD disease progression. Based on this hypothesis, studies and clinical trials have been carried out to evaluate nilvadipine, a calcium channel blocker for the treatment of hypertension, for delaying AD disease progression [24,25]. Despite nilvadipine’s conflicting clinical study results in treating AD, it is still accepted that hypertension promotes the pathogenesis of AD, and reducing hypertension before or during AD onset may help slow down AD disease progression [24,25,26,27].

The discovery of artemisinin is a phenomenal example to demonstrate that traditional Chinese medicine (TCM) is an invaluable source for the modernization of conventional drugs [28,29]. Much effort has been made to understand the complex system of TCM, with a combination of its traditional treatments and modern technological means [30,31,32]. Network pharmacology is a branch of system biology that facilitates drug identification and design for multi-target compounds by selecting the key genes and their biological processes and functional pathways. It integrates and visualizes the complex networks among compounds, targets, disease targets, and biological processes [33]. With the help of network pharmacology, it is possible to reveal the intricate molecular mechanisms underlying TCM and further establish novel therapeutic application beyond the traditional TCM application. For example, in combination with a strategy using high-throughput sequencing technique, Li S. et al. screened 22 health-strengthening and 25 pathogen-eliminating herbs to identify potential target profiles of 1446 TCM compounds, and several cancer-related pathways of those compounds were identified, such as apoptosis, vascular endothelial growth factor (VEGF) signaling pathway, T cell receptor related pathways, etc. Those herbs have a more significant regulatory role in immune and tumor microenvironment, rather than direct cytotoxic effects on tumors [34]. As for exploring novel TCM applications using network pharmacology, Li JY et al. combined network pharmacology, machine learning, deep learning, and molecular dynamics simulation to investigate potent lead drugs to identify insulin-like growth factor 1 receptor (IGF1R) and insulin receptor (IR) as two key genes, as well as three potential longevity-related herbal drugs from the established TCM databases [35]. It is known that IGF1R and IR are related to tumor growth and insulin insensitivity, respectively. These results indicate that the three longevity-related herbs have novel therapeutic potentials in cancer treatment and type II diabetes in a novel or improved TCM formulae. The above examples show that studies on this scale are impossible to perform using traditional laboratory techniques and resources, but that network pharmacology makes it possible and illuminates directions for future studies. Therefore, network pharmacology can help optimize research design, establishing novel clinical applications, and improving cost efficiency, which will be beneficial to the research and advance of TCM.

In this study, we use network pharmacology to screen the *Uncaria* alkaloids that may alleviate AD symptoms via working on the cardiovascular system and also to find their potential targets and molecular mechanisms. To better understand the mechanisms and targets of these alkaloids, the in-house library of *Uncaria* alkaloids was compiled from our previous study. An integrated analytical platform was built based on network pharmacology, including target prediction, protein–protein interaction (PPI) network, topology analysis, gene enrichment analysis, and molecular docking. By combining this platform and biological experiments, we explored the underlying mechanisms of *Uncaria* alkaloids’ anti-hypertensive effects and explored the possible application of *Uncaria* alkaloids in preventing AD.

## 2. Results

The schematic workflow of this study is demonstrated in the graphical abstract.

### 2.1. The In-House Library of Uncaria Alkaloids and Drug-Likeness Screening

One hundred and forty-three *Uncaria* alkaloids from *Uncaria* genus were included in the in-house compound library for drug-likeness analysis using the FAFDrugs4 program. From 143 compounds, 83 were accepted as candidate compounds (Table S1). Out of the rejected compounds, 3-dihydrocadambine (546.6 g/mol) and vincosamide (498.5 g/mol) were rejected because their predicted numbers of rigid bonds and max ring size exceeded the threshold. They were included in the library based on their reported bioactivities from the literature search or previous studies [4,36,37]. Therefore, a total of 85 *Uncaria* alkaloids were used in the following target prediction.

### 2.2. Putative Target Prediction for the Candidate Targets of Uncaria Alkaloids

**Figure 1:**
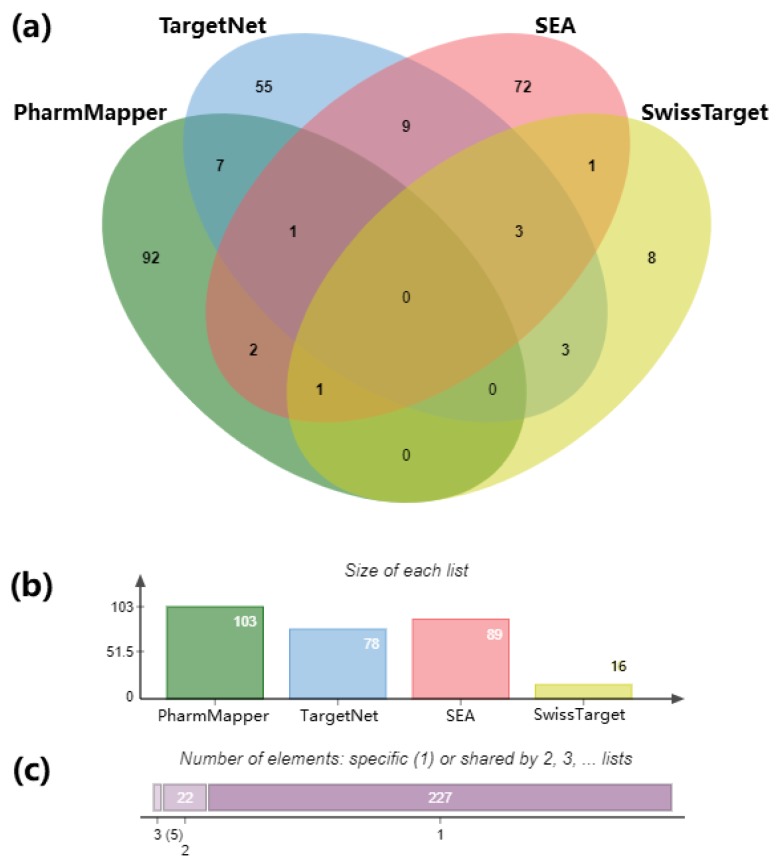
Analysis of the predicted genes for the 85 *Uncaria* alkaloids from four databases. (**a**) Venn graph showing the numbers of the overlapped and the specific genes predicted from PharmMapper (green), TargetNet (blue), SEA (pink), and SwissTarget (yellow); (**b**) numbers of predicted genes from each database; (**c**) a summary of numbers of genes shared among the databases: 227 specific genes, 22 genes shared by two databases, and five genes found in three databases.

To find the target genes of the 85 *Uncaria* alkaloids, PhamaMapper, TargetNet, SEA and SwissTarget servers were used to predict the *Uncaria* alkaloids’ compound targets, and the numbers of overlapped targets were visualized in Figure 1a. There were 103 targets from PharmMapper (Norm fit > 0.8), 78 targets from TargetNet server (probability > 0.8), 89 targets identified in SEA database, and 16 from SwissTarget (probability > 0.5) (Figure 1b). No shared targets were found at the intersection of all four databases. Overall, there are 227 specific genes, 22 genes shared by two databases, and only five genes found in three databases (Figure 1c). After removal of the partially overlapped targets, 254 genes were found in the union, and they were selected as the potential targets of *Uncaria* alkaloid for the following analysis. The 254 genes and their functional annotations were visualized using ClueGO software (Figure S1).

### 2.3. Identification of Approved Druggable Targets in AD and Hypertension

**Figure 2:**
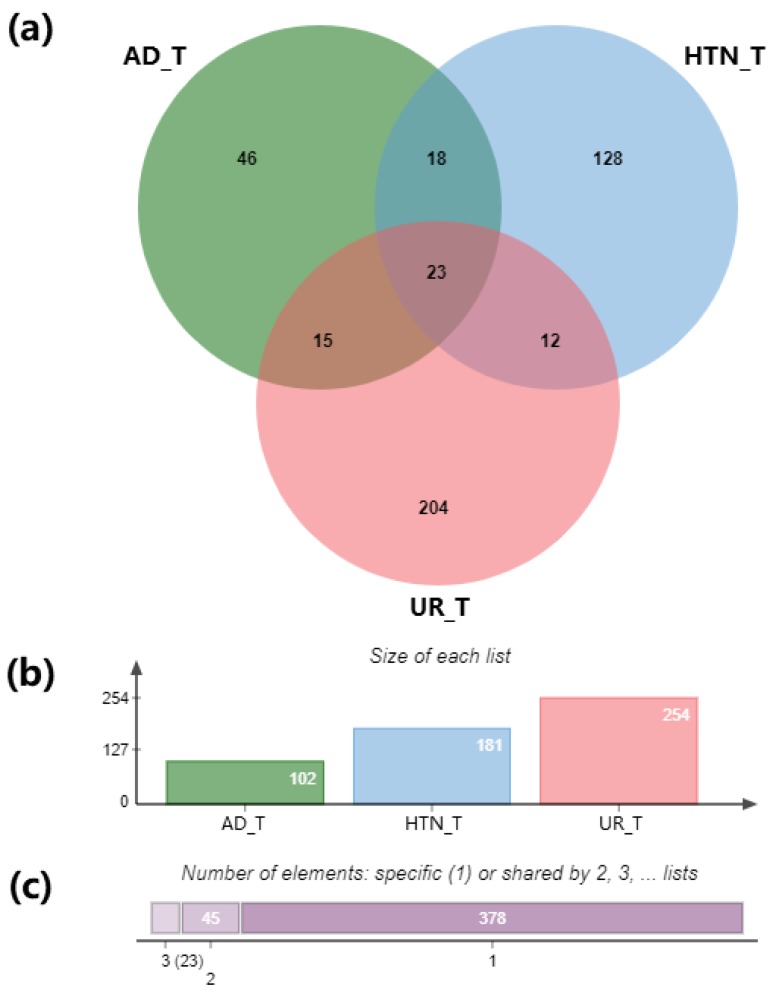
Analysis of the predicted compound-disease target genes among the 85 *Uncaria* alkaloids (UR_T), Alzheimer’s disease (AD_T), and hypertension (HTN_T). (**a**) Venn graph showing the numbers of the overlapped and the specific genes among AD, HTN, and UR; (**b**) numbers of predicted genes from each set; (**c**) a summary of numbers of genes shared among the gene sets: 378 specific genes, 45 genes shared by two sets, and 23 genes found in all three sets of genes.

DrugBank and TTD databases were used to identify the approved therapeutic targets of AD and hypertension, and the overlap of the results was visualized in the venn graph (Figure 2a). After redundancy removal, 102 therapeutic targets were identified for AD and 181 for hypertension (Figure 2b). When overlapped with the 254 targets of *Uncaria* alkaloids, twenty-three targets were found in the intersection of *Uncaria* alkaloids, AD, and hypertension (Figure 2a).

### 2.4. Pathways in Compound-Target Network

**Figure 3:**
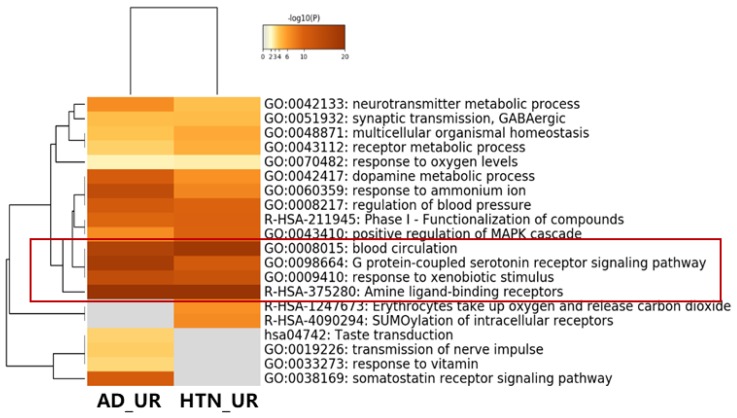
Heatmap of pathway analysis between AD_UR and HTN_UR using Metascape database. Targets in AD_UR and HTN_UR shared important biological processes, such as “amine ligand-biding receptors”, “response to xenobiotic stimulus”, “blood circulation”, and “G protein-coupled serotonin receptor signaling pathway”.

To verify the shared targets among *Uncaria* alkaloids (UR) and the disease targets from Alzheimer’s disease (AD) and hypertension (HTN), the 38 shared targets in “AD_UR” and the 35 shared targets in “HTN_UR” were analyzed using MetaScape (Figure 2a and Figure 3). The heatmap of GO analysis showed that “amine ligand-binding receptors”, “response to xenobiotic stimulus”, “blood circulation”, and “G protein-coupled serotonin receptor signaling pathway” are important biological processes shared by AD_UR and HTN_UR (Figure 3).

### 2.5. Network Construction, Topological Analysis, and GO/KEGG Function and Pathway Analysis

**Figure 4:**
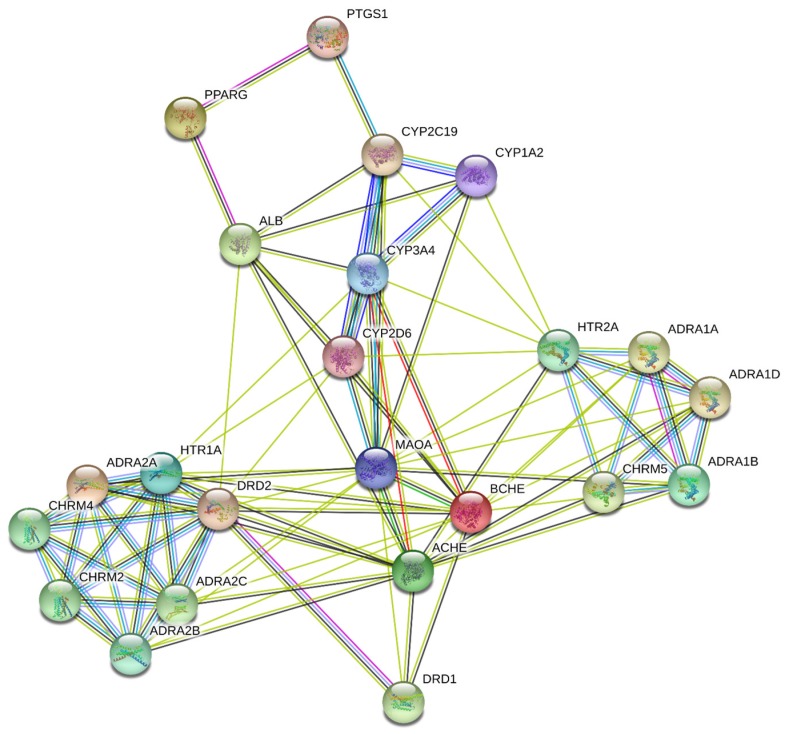
Visualization of the protein–protein interaction (PPI) of the 23 target genes* using STRING and Cytoscape databases.

All genes were presented in HGNC (HUGO Gene Nomenclature Committee) gene symbol format in the following analysis to avoid confusion across databases and platforms. The interaction between the 23 genes was analyzed and visualized using STRING and Cytoscape databases. Under “medium confidence (0.4 by default)”, the protein–protein interaction (PPI) network was constructed (Figure 4). The results and parameters from the PPI topological analysis are listed in Table 1. MAOA (monoamine oxidase A), ACHE (acetylcholinesterase), BCHE (butyrylcholinesterase), DRD2 (dopamine receptor D2), and HTR1A (serotonin 1A receptor) were the top 5 genes based on degree (Table 1, Column 3). The scores of other parameters are listed in Table 1 (Columns 4–10)—no significant alterations were found when the target ranks were based on other parameters (Table 1).

### 2.6. Pathways in Target-Disease Network

**Figure 5:**
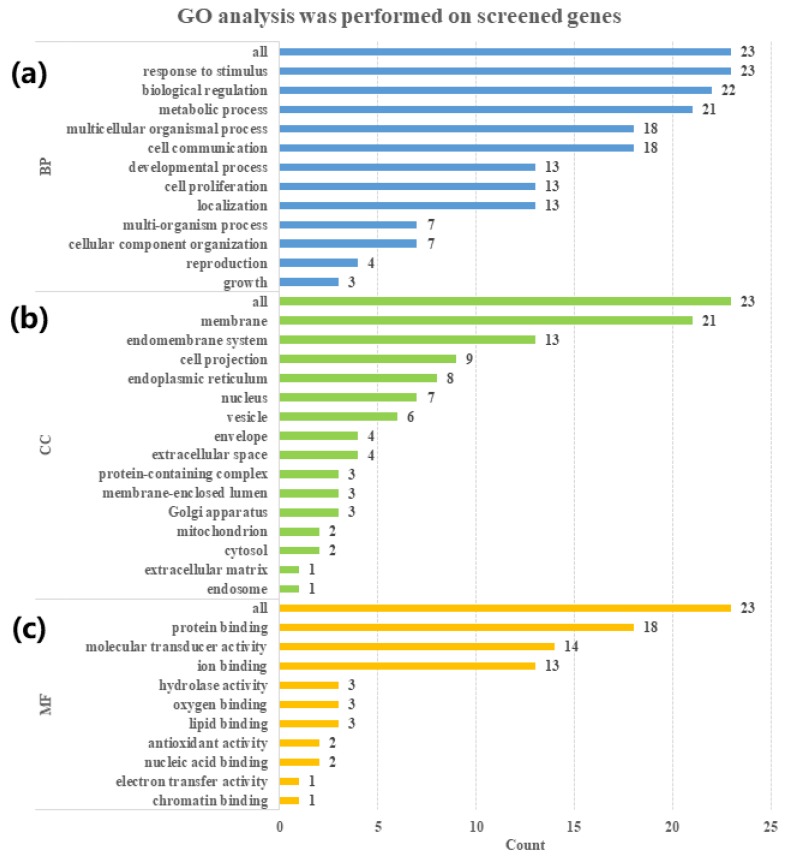
GO analysis of target–disease gene interactions for *Uncaria* alkaloids to reveal the related (**a**) biological process (BP); (**b**) cellular component (CC); and (**c**) molecular function (MF). The numbers of genes included are marked next to the horizontal bar graph.

The list of the 23 screened genes was uploaded to the WebGestalt database for GO enrichment analysis. Three categories, i.e., biological process (BP), cellular component (CC), and molecular function (MF), are presented in Figure 5. The 23 targets were involved in many biological processes including “response to stimulus”, “biological regulation”, “metabolic process”, and “cell communication” (Figure 5a). “Membrane” and “endomembrane system” ranked the highest in the cellular component category (Figure 5b), while “protein binding”, “molecular transducer activity”, and “ion binding” were the primary molecular function involved (Figure 5c).

### 2.7. Pathways in Target-Pathway Network

**Figure 6:**
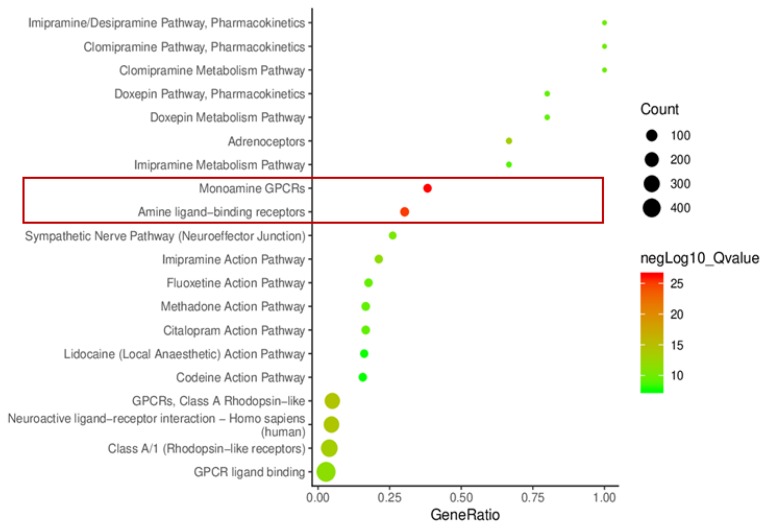
Bubble graph demonstrating statistically significant pathways that include the 23 genes analyzed using the ConsensusPathDB-human database. The results showed that “amine ligand-binding receptors” and “monoamine GPCRs” are two important pathways regarding *Uncaria* alkaloids, AD, and hypertension.

**Figure 7:**
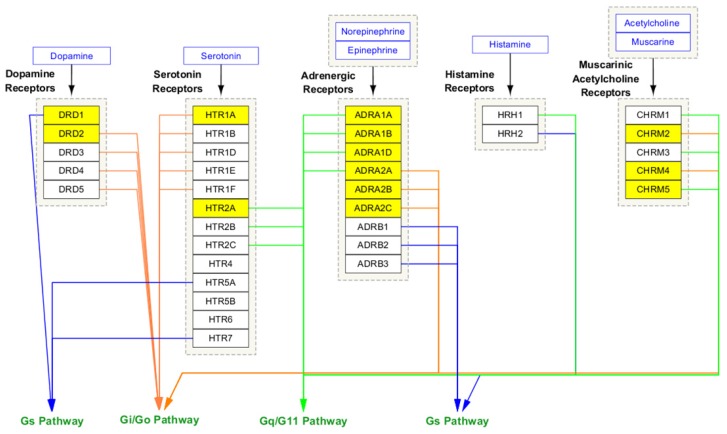
Detailed analysis of the 13 out of the 23 screened genes in “monoamine GPCRs” pathway. The 13 genes are highlighted, and they are distributed in categories of adrenergic receptors, dopamine receptors, serotonin receptors, and muscarinic acetylcholine receptors, but not in histamine receptors. Arrows: blue, Gs pathway; orange, Gi/Go pathway; green, Gq/G11 pathway.

Using the ConsensusPathDB-human database, the top 20 pathways of the 23 predicted genes are listed in Figure 6—the two pathways that showed statistical significance are “monoamine GPCRs” and “amine ligand-binding receptors” and are represented by the two red dots in the bubble graph (Figure 6). There were 13 out of the 23 screened genes present in the “monoamine GPCRs” pathway. More detailed pathway information of the 13 genes is highlighted in Figure 7, showing that they are distributed in categories of adrenergic receptors, dopamine receptors, serotonin receptors, and muscarinic acetylcholine receptors, but not in histamine receptors (Figure 7).

### 2.8. Integrative Network Analysis and Target Selection

**Figure 8:**
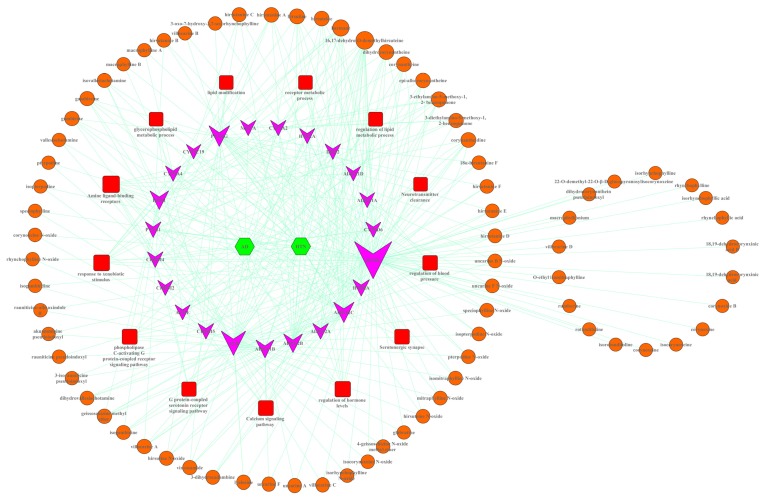
Comprehensive representation of the built network of *Uncaria* alkaloids, AD, and hypertension. Diseases are represented by hexagons (green), target genes by arrowheads (magenta), *Uncaria* alkaloids by circles (orange), and pathways by squares (red).

To further obtain a comprehensive understanding between the selected *Uncaria* alkaloids, the selected target genes, and the diseases, an integrative network analysis was performed using Cytoscape (Figure 8). An intricate network was formed among the *Uncaria* alkaloids and their potential targets regarding AD and hypertension.

### 2.9. Molecular Docking and Biological Validation

**Figure 9:**
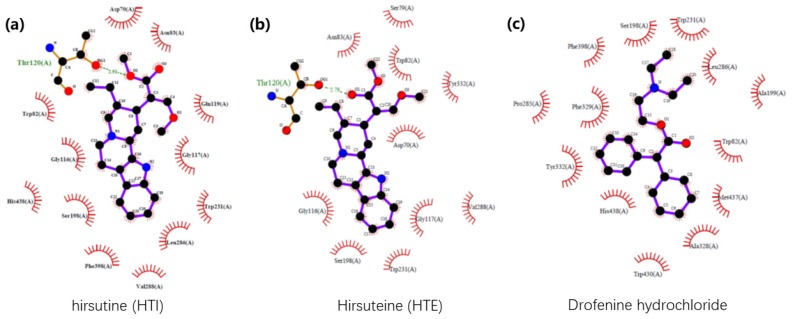
Schematic diagrams demonstrating the butyrylcholinesterase (BChE) binding sites and proximate affinity of hirsutine (HTI), hirsuteine (HTE) and drofenine hydrochloride (a BChE inhibitor). Black dots: carbon atoms; blue dots: nitrogen atoms; red dots: oxygen atoms; green dotted lines: hydrogen bonds; red combs: amino acid residues.

Among the top six genes in Table 1, there were 74 *Uncaria* alkaloids identified for BCHE. Only four *Uncaria* alkaloids were corresponding to the other five genes (MAOA, ACHE, DRD2, HTR1A, and HTR2A) across various databases. Therefore, BCHE was chosen as a more efficient target for the following biological validation (see Raw data.elsx). To validate the ability of *Uncaria* alkaloids to target the selected genes, we chose BChE (PDB ID: 4TPK) as an example for molecular docking. The scores are listed in Table 2. As shown, hirsuteine (HTE) and hirsutine (HTI) were the two compounds with the highest scores of 6.7146 and 6.4769, respectively (Table 2). Their binding sites and affinity (2D ligand–protein interaction diagrams) are also shown in Figure 9. Galantamine hydrobromide, a selective AChE inhibitor, had a lower score of 4.8609, while the BChE inhibitor drofenine hydrochloride had the highest score of 9.7639.

**Figure 10:**
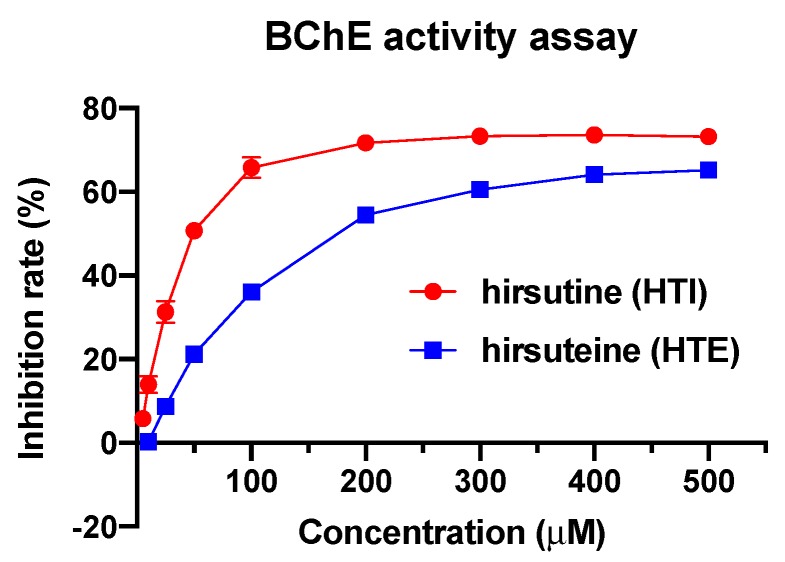
Inhibition rate of hirsutine (HTI) and hirsuteine (HTE) in BChE activity assay. HTI IC_50_ = 67.97 μM; HTE IC_50_ = 202.3 μM.

The biological effects of the selected *Uncaria* alkaloids were further evaluated using BChE enzyme activity assay, with HTE and HTI showing significant inhibitory effects on BChE activity. The IC_50_ of each compound was 67.97 μM and 202.3 μM (Table 3 and Figure 10). Geissoschiziner, corynoxeine and isocorynoxeine showed moderate inhibitory effects on BChE at a high concentration of 500 μM (Table 3). No inhibitory activity was detected in rhynchophylline, isorhynchophylline, 3-dihydrocadambine, or vincosamide (data not shown).

## 3. Discussion

As one of the representative herbs of traditional Chinese medicine (TCM), the *Uncaria* genus has received extensive investigation in modern pharmacology due to its effects on alleviating symptoms related to the cardiovascular system and central nervous system (CNS) [1,2,3,5,7]. To better understand the mechanisms behind the clinical applications of *Uncaria*, we built an integrated analytical platform based on network pharmacology, including target prediction, protein–protein interaction (PPI) network, topology analysis, gene enrichment analysis, and molecular docking. Using this platform, we identified some underlying mechanisms of anti-hypertensive effects and explored the possibility of *Uncaria* alkaloids in preventing Alzheimer’s disease (AD). The butyrylcholinesterase (BChE) enzyme activity assay provided further supports for the results from the network analysis and molecular docking, showing the feasibility of the current analytical approach. Therefore, using *Uncaria* as an example, this study provides a novel strategy for understanding the holistic pharmacology of TCM, as well as for exploring the multi-indication property of TCM beyond its traditional applications.

*Uncaria* genus can be found in many ancient TCM formulas treating hypertension, and *Uncaria* alkaloids are considered as the major bioactive compounds [1,4,5]. For example, rhyncophylline and isorhyncophylline, two of the most widely studied indole alkaloids from the *Uncaria* genus, can inhibit the neuronal release of catecholamines, block the conduction between neurons, and indirectly achieve anti-hypertensive effects [7,38,39,40,41]. Further evidence indicates that the cardiovascular regulation of rhyncophylline and isorhyncophylline comes from direct vasodilation, down-regulation of cardiac output, and reduction of calcium influx [1,7,42]. All these actions cause vasodilation and lead to the reduction of blood pressure, yet the exact mechanisms are still to be elucidated.

Like in other systems of the human body, vascular impairment in the brain causes further tissue and organ damage, usually in the form of chronic neuroinflammation, which can lead to neurodegenerative diseases [15,16,17,18,20,21]. Though age and genetics contribute a significant part to AD pathogenesis, hypertension is also generally accepted as one of the significant risk factors for AD, together with other risk factors including diabetes mellitus, head trauma, and depression [17,43,44,45]. Ye et al. revealed that rhyncophylline has therapeutic potentials in alleviating AD via targeting the EphA4 receptor [46]. Other studies on *Uncaria* and AD have shown that *Uncaria* alkaloids have neuroprotective effects on reducing beta-amyloid aggregation and deposition [6,8,47]. From the PPI network and topological analysis in our study, several possibly synergic and promising effects of *Uncaria* have been revealed for the slowing down, if not blocking or reversing, of AD pathogenesis (Figure 5 and Table 1). The “multiple mechanisms and multitargets” property of *Uncaria*’s effects on AD is also no exception to the characteristic pharmacological effects of natural products, which often demand a more comprehensive and integrative analytical strategy to study.

In 2002, Dr. Shao Li and his team elaborated on the non-linear, open, and complex properties of TCM from a symptomatological, network, and systemic perspective [30,31,48]. British pharmacologist Dr. Andrew L. Hopkins established the concept of “network pharmacology”, and defined it as a subdivision of pharmacology, which can analyze the overall effects and interactions among the multiple compounds, their targets, and the related pathways [49,50]. With the help of network pharmacology, significant progress has been made in understanding the complicated and complex systems such as the pharmacology of TCM formula and compositions [34,51,52,53,54].

During the analytical workflow of network pharmacology, the critical step is the prediction and selection of the targets. Currently, two common approaches are available, one based on the ligands and the other based on the receptors for drug design, drug discovery and evaluation [55,56]. It can also be classified into four categories: 2D/3D structure similarity, computer-aided Quantitative Structure-Activity Relationship (QSAR), pharmacophore, and molecular docking [57,58]. Both advantages and disadvantages of the four approaches exist, and their prediction accuracy usually improves when combining two or more approaches.

Therefore, in the current study, three out of the four approaches were adopted, including the conformation-, QSAR- and pharmacophore-based approaches (Figure 1). Based on the three approaches, we built the “compound-targets-pathways” network. The analysis predicted that *Uncaria* alkaloids may affect “monoamine GPCRs”, “amine ligand-binding receptors”, “response to xenobiotic stimulus”, “blood circulation”, and “G protein-coupled serotonin receptor signaling pathway” to achieve the dual benefits in treating hypertension and alleviating AD at the same time (Figure 4 and Figure 7). When the predicted results were further evaluated, BChE—one of the key targets related to the “response to xenobiotic stimulus” pathway—was picked for further validation. As a promising druggable target for AD, BChE activity is significantly elevated in later stages of AD [59,60,61]. Our study indicates that BChE may be a shared target for both AD and hypertension (Table 1). Hirsutine and hirsuteine—two *Uncaria* alkaloids from *Uncaria rhynchophylla*—showed a significant inhibitory effect on BChE activity (Figure 10). These results are consistent with the reported pharmacological effects of *Uncaria* extracts, and they demonstrated the efficiency and accuracy of our analytical platform on *Uncaria* alkaloids [6,9].

One of the limitations of this study is that the results from molecular docking cannot directly indicate the excitatory and inhibitory effects on the selected targets. Therefore, biological validation is usually performed to help evaluate and refine the results from the network pharmacology. Besides, the biological validation only covers a few selected *Uncaria* alkaloids with higher abundance. Therefore, the spectrum and potential of *Uncaria* alkaloids in treating AD and hypertension demonstrated here cannot be identical to the full effects of *Uncaria* plant extracts used clinically as a herbal medicine.

One more aspect to consider is the bioavailability of hirsutine and hirsuteine in vivo. One study from 2013 states that hirsutine and hirsuteine can be detected in rat plasma after oral administration of a herbal formula containing *Uncaria* hooks [62]. Another study found the bioavailability of hirsutine and hirsutine via intravenous delivery is at 4.4% and 8.21%, respectively [63]. The pharmacokinetic (PK) properties of hirsutine, hirsuteine, and 3-dihydrocadambine (DHC) were also measured in our laboratory [64]. The AUC_last_ (hr*ng/mL) of the three alkaloids were 148.29 ± 54.11, 1369.78 ± 736.15, and 275.27 ± 52.19, respectively [64]. Taking DHC as an example, despite its low bioavailability, our laboratory shows that DHC has a potent anti-hypertensive effect, which is consistent with a study by Aisaka et al. in 1985 [36]. Based on these studies, it is evident that there is a conflict between the low bioavailability of *Uncaria* alkaloids and their effectiveness in treating CNS symptoms (interpreted as “mood calming and mild sedation” based on *Uncaria*’s traditional application). It indicates that more analysis and in depth discussion are necessary to study the distribution and bioavailability of *Uncaria* alkaloids in the brain in our future studies.

In summary, our study has visualized an intricate network among *Uncaria* alkaloid compounds, their potential disease targets of AD and hypertension, and the possible pathways and biological processes. Though more biological validation is needed to further validate the current results, for the first time, the possibility of *Uncaria* alkaloids in delaying AD disease progression has been explored from the perspective of blood pressure control in a systemic approach. This combination of TCM and modern analytical methods may shine a new light on our approaches to studying TCM and provide a new therapeutic strategy and targets for patients who suffer from hypertension and AD.

## 4. Materials and Methods

### 4.1. Compound Library Construction and Drug-Likeness Screening

The in-house library of *Uncaria* alkaloids was compiled from previously published data from our laboratory (Table S1). *Uncaria* alkaloid compounds included in the library were first screened for their drug-likeness analysis using the FAFDrugs4 database [65]. The FAFDrugs4 database generates a computational prediction of drug likeness of the compounds to facilitate the drug-likeness screening. Duplicates and rejected molecules were discarded from the library, and only accepted compounds were included in the following analysis. Additional compounds were added back to the compound library because of their reported bioactivity from our earlier study [4]. Detailed information of the in-house library and accepted compounds is listed in the Supplementary Excel file.

### 4.2. Prediction of Uncaria Alkaloid Targets and Their Overall Biological Functions

All the information about the applied software, databases, and servers is listed in Table S2. Putative target prediction of the *Uncaria* alkaloids was made using an integrative application of TargetNet, SwissTargetPrediction, SEA, and PharmMapper servers (Table S2) [56,66,67,68,69,70]. These databases adopted various prediction algorithms and generated compound targets (genes). All the genes were presented in HGNC (HUGO Gene Nomenclature Committee) gene symbol format in the following analysis to avoid confusion across databases and platforms. These targets were pooled, and entries with “homo sapiens” origin were included in the following analysis. The overlaps were drawn using jvenn [71].

The above compound targets were further analyzed using the ClueGo plug-in of Cytoscape, and the overall protein–protein interaction (PPI) within the 85 *Uncaria* alkaloids and their targets were depicted (Table S2) [72,73].

### 4.3. Identification of Druggable Alzheimer’s Disease (AD) and Hypertension (HTN) Associated Disease Targets

To compile the disease targets for AD and hypertension, we searched with the keywords “Alzheimer’s disease” and “high blood pressure”/“hypertension” respectively in both DrugBank and TTD databases (Table S2) [74,75]. Only “approved” drugs with no limitations in “market availability” were included for the disease-related targets. For each disease, duplicated drug targets (genes) were removed. The overlapped and special targets in each target set were visualized using jvenn [71].

### 4.4. Network Construction, Topological Analysis and GO/KEGG Function Analysis

The “compound-disease targets” were the intersection of *Uncaria* alkaloid compound targets, AD disease targets, and HTN disease targets. In order to elucidate the interconnection between *Uncaria* alkaloid compounds, their potential target in AD and hypertension diseases, and the underlying biological mechanisms, a protein–protein interaction (PPI) network was constructed and analyzed with STRING and Cytoscape platforms (Table S2) [73,76]. The topological properties of every node in the interaction network were analyzed, and the results were ranked by “degree”, betweenness centrality (BC), degree centrality (DC), eigenvector centrality (EC), closeness centrality (CC), network centrality (NC), and local average connectivity (LAC). Nodes (targets) with higher ranks were considered to have a more critical role within the network.

To further find out the biological functions within the constructed network, GO/KEGG analyses for the compound-target network, target-disease network, and target-pathway network were performed with MetaScape, WebGestalt, and ConsensusPathDB respectively [77,78,79]. To analyze and visualize the GO/KEGG function, a GO Enrichment plot was created using ImageGP (http://www.ehbio.com/ImageGP/). The enriched target-pathway information was further visualized by WikiPathways database [80].

### 4.5. Molecular Docking

Molecular docking was performed with SYBYL-X Software (Version 2.0, Tripos International, St. Louis, MO, USA) using BCHE as a demonstration. 3D molecular structure was obtained from Pubchem website or drawn with ChemDraw (Version 19.0, PerkinElmer Informatics, Waltham, MA, USA), and then imported to SYBYL-X 2.0 for energy and conformation optimization with the following settings: “method: powell; termination: gradient; maximum iterations: 1000; force field: MMFF94; other parameters: default” [81]. Optimized small molecules were saved as mol_2_ format. A Surflex-Dock approach was applied to analyze and repair the molecular structure of BCHE (RCSB PDB ID: 4TPK), and the docking targets were further defined [82]. The mol_2_ file with information about the small molecules was applied to perform molecular docking on BCHE. The scores were collected for further analysis, and the results are summarized in Table 1.

### 4.6. In Vitro Butyrylcholinesterase (BChE) Enzyme Activity Assays

The enzymes BChE (Sigma-Aldrich, St. Louis, MO, USA) were dissolved in ultrapure water to make the 20 unit/mL stock solution, and the enzyme solutions were stored at −80 °C. The enzyme stock solution was diluted with 50 mM Tris-HCl (pH 7.8) before use. Solutions of tested compounds were prepared to start from 500 M stock solutions in dimethyl sulfoxide (DMSO; Thermo, Rockford, IL, USA) and diluted to a series of concentrations before the experiment. 5,5′-dithiobis-bis-nitrobenzoic acid (DTNB; Sigma-Aldrich, St. Louis, MO, USA) and butyrylthiocholine iodide (BTCI; Sigma-Aldrich, St. Louis, MO, USA) needed to be freshly dissolved in phosphate buffer (pH 7.6). The inhibitory activity of these *Uncaria* alkaloids against BChE was carried out in vitro using the Ellman colorimetric method with minor modifications [83]. Briefly, BTCI was used as substrate and DTNB as the chromophoric reagent. One hundred and forty μL phosphate buffer solution (pH 7.6), 20 μL 0.5 u/mL BChE, and 20 μL test compounds solution were added to the 96-well plate in turn. After being mixed and pre-incubated for 15 min at 25 °C, 10 μL 750 mM BTCI and 10 μL 500 mM DTNB were added to the mixture to start the reaction. After incubation for 30 min at 37 °C, the spectrophotometric absorption at 405 nm on an 800™ TS Absorbance Reader (Bio-Tek, Winooski, VT, USA) was measured immediately. 20 μL phosphate buffer solution (pH 7.6) was added to the blank group instead of the compounds to be tested. Galantamine (Selleck-China, Shanghai, China), a general cholinesterase inhibitor and FDA approved drug for AD treatment, was used as the positive control. The results of galantamine in the BChE activity assay was included in the Supplementary Materials (Table S3). All measurements were conducted in triplicate. Data were presented as mean ± S.D., and the IC_50_ values were graphically calculated from the inhibition curves by GraphPad Prism (Version 5.00, Graphpad Software, La Jolla, CA, USA).

## Figures and Tables

**Table 1:** Topological analysis of the PPI network of the 23 genes shared by *Uncaria* alkaloids, AD and hypertension. Data were ranked by degree.

#	Gene Symbol	Degree	Subgraph	Eigenvector	Information	LAC	Betweenness	Closeness	Network
1	MAOA	16	1850.135	0.356	5.360	6.375	81.651	0.759	13.078
2	ACHE	14	1413.283	0.311	5.162	5.143	63.508	0.710	9.577
3	BCHE	13	1311.436	0.300	5.048	4.923	38.836	0.688	8.028
4	DRD2	12	1230.716	0.290	4.924	6.333	35.935	0.667	9.991
5	HTR1A	11	1166.267	0.282	4.786	6.545	20.340	0.629	9.071
6	HTR2A	10	623.717	0.203	4.632	4.800	24.033	0.595	7.526
7	ADRA2B	9	868.194	0.242	4.461	6.444	7.798	0.595	8.000
8	ADRA2C	9	868.194	0.242	4.461	6.444	7.798	0.595	8.000
9	ADRA2A	9	868.194	0.242	4.461	6.444	7.798	0.595	8.000
10	CYP3A4	9	688.133	0.216	4.461	4.889	10.826	0.629	6.405
11	ALB	8	406.076	0.165	4.268	2.750	47.899	0.611	3.429
12	CYP2D6	8	606.406	0.203	4.268	4.500	9.607	0.611	5.238
13	ADRA1D	7	428.160	0.169	4.049	4.857	1.567	0.537	6.000
14	ADRA1B	7	428.160	0.169	4.049	4.857	1.567	0.537	6.000
15	ADRA1A	7	428.160	0.169	4.049	4.857	1.567	0.537	6.000
16	CYP2C19	7	271.666	0.133	4.049	3.429	39.552	0.564	4.333
17	CHRM2	6	348.284	0.151	3.798	5.000	0.000	0.458	6.000
18	CHRM4	6	348.284	0.151	3.798	5.000	0.000	0.458	6.000
19	CHRM5	5	178.487	0.107	3.508	4.000	0.000	0.512	5.000
20	CYP1A2	5	193.747	0.112	3.508	3.200	1.269	0.537	4.000
21	DRD1	4	252.070	0.131	3.170	2.500	0.167	0.524	3.333
22	PPARG	2	7.872	0.019	2.287	0.000	2.700	0.400	0.000
23	PTGS1	2	6.536	0.016	2.287	0.000	1.583	0.379	0.000

**Table 2:** Selected *Uncaria* alkaloids and their total scores from molecular docking analysis using SYBYL-X 2.0 Software.

Ligand	Total Scores
3F9_A	5.524
3F9_B	4.6765
Corynoxine B	5.7701
Corynoxeine (C)	5.4715
Corynoxine	5.58
3-dihydrocadambine (DHC)	5.0741
Geissoschiziner (GSM)	5.886
Hirsuteine (HTE)	6.7146
Hirsutine (HTI)	6.4769
Isocorynoxeine (IC)	6.196
Isorhynchophylline (IR)	6.1038
isomitraphylline	4.3782
Rhynchophylline (R)	4.8015
Uncarine B	4.8631
Vincosamide (VCA)	5.9499
Galantamine hydrobromide	4.8609
Drofenine hydrochloride	9.7639

**Table 3:** In vitro BChE enzyme activity assay of five selected *Uncaria* alkaloids.

Conc. (μM)	Inhibition Rate (%)				
	Hirsutine (HTI)	Hirsuteine (HTE)	Geissoschiziner (GSM)	Corynoxeine	Isocorynoxeine
50	50.71 ± 1.51	21.23 ± 0.74	0.07 ± 0.99	-	-
100	65.83 ± 2.49	36.03 ± 0.67	7.34 ± 0.66	-	-
200	71.69 ± 0.18	54.44 ± 0.92	22.24 ± 1.32	(−1.86) ± 1.65	(−1.86) ± 0.55
300	73.28 ± 0.67	60.51 ± 0.74	n.a.	1.76 ± 0.98	3.09 ± 1.58
400	73.60 ± 0.67	64.13 ± 0.18	n.a.	16.82 ± 3.39	15.17 ± 3.50
500	73.17 ± 1.15	65.19 ± 0.96	39.83 ± 1.52	19.43 ± 3.21	21.77 ± 0.90
IC_50_	67.97	202.3	n.a.	n.a.	n.a.

## Supplementary Materials

Supplementary materials can be found at https://www.mdpi.com/1422-0067/21/5/1766/s1.

## Abbreviations

AD	Alzheimer’s disease
BChE	butyrylcholinesterase
HTN	hypertension
PPI	protein–protein interaction
TCM	Traditional Chinese Medicine
