# Supplementary material for: A Network-Based Approach to Explore the Mechanisms of Uncaria Alkaloids in Treating Hypertension and Alleviating Alzheimer’s Disease
Source: Int J Mol Sci. 2020 Mar 4;21(5):1766. doi: 10.3390/ijms21051766 (PMC7084279; doi:10.3390/ijms21051766)
Supplement: Supplementary file 1 [file ijms-21-01766-s001.zip › ijms-723574 supplementary done/Supplementary Files/Supplementary material.pdf]

Supplementary Material:

A network-based approach to explore the mechanisms of Uncaria alkaloids in treating hypertension and alleviating Alzheimer's disease

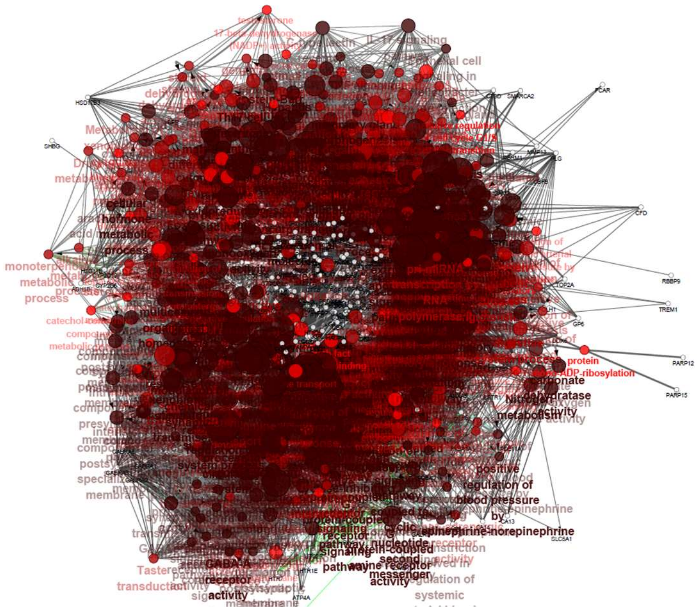

Figure S1. Network of compound-target gene interactions for Uncaria alkaloids built by linking the identified 85 compounds and 254 target genes using ADME screening.

Table S1 List of the 143 Uncaria alkaloids isolated from Uncaria genus

No.	Compound name	Molecular formula	Molecular weight	References
**β-carboline type**				
1	harmane	$C_{12}H_{10}N_2$	182.0844	[1]
2	lyaloside	$C_{27}H_{30}O_9N_2$	526.1951	[2]
3	deoxycordifoline	$C_{28}H_{30}O_{11}N_2$	570.1850	[3]
4	5β-carboxystrictosidine	$C_{28}H_{34}O_{11}N_2$	574.2163	[2]
5	3,4-dehydro-5-carboxystrictosidine	$C_{28}H_{32}O_{11}N_2$	572.2006	[2]
6	hirsutaside A	$C_{30}H_{38}O_{11}N_2$	602.2476	[4]
7	hirsutaside D	$C_{43}H_{52}O_{18}N_2$	884.3215	[5]
8	neonaucleoside B	$C_{45}H_{54}O_{20}N_2$	942.3270	[5]
9	bahienoside A	$C_{44}H_{58}O_{18}N_2$	902.3685	[5]
10	bahienoside B	$C_{44}H_{58}O_{18}N_2$	902.3685	[5]
**tetracyclic monoterpene oxindole type, tMOA**				
11	corynoxeine	$C_{22}H_{26}O_4N_2$	382.1893	[6]
12	isocorynoxeine	$C_{22}H_{26}O_4N_2$	382.1893	[6]
13	corynoxine	$C_{22}H_{28}O_4N_2$	384.2049	[7]
14	corynoxine B	$C_{22}H_{28}O_4N_2$	384.2049	[8]
15	18,19-dehydrocorynxinic acid	$C_{21}H_{24}O_4N_2$	368.1736	[9]
16	18,19-dehydrocorynxinic acid B	$C_{21}H_{24}O_4N_2$	368.1736	[9]
17	rhynchophyllic acid	$C_{21}H_{26}O_4N_2$	370.1893	[10]
18	isorhynchophyllic acid	$C_{21}H_{26}O_4N_2$	370.1893	[10]

19	rhynchophylline	$C_{22}H_{28}O_4N_2$	384.2049	[6]
20	isorhynchophylline	$C_{22}H_{28}O_4N_2$	384.2049	[6]
21	22-*O*-demethyl-22-*O*-*β*-D-glucopyranosylisocorynoxeine	$C_{27}H_{34}O_9N_2$	530.2264	[11]
22	corynoxeine *N*-oxide	$C_{22}H_{26}O_5N_2$	398.1842	[11]
23	isocorynoxeine *N*-oxide	$C_{22}H_{26}O_5N_2$	398.1842	[11]
24	rhynchophylline *N*-oxide	$C_{22}H_{28}O_5N_2$	400.1998	[11]
25	isorhynchophylline *N*-oxide	$C_{22}H_{28}O_5N_2$	400.1998	[11]
26	dihydrocorynanthein pseudoindoxyl	$C_{22}H_{28}O_4N_2$	384.2049	[1]
27	macrophyllionium	$C_{23}H_{30}O_4N_2$	398.2206	[7]
28	macrophylline A	$C_{25}H_{32}O_5N_2$	440.2311	[7]
29	macrophylline B	$C_{25}H_{32}O_5N_2$	440.2311	[7]
30	villocarine C	$C_{22}H_{26}O_5N_2$	398.1842	[12]
31	villocarine D	$C_{22}H_{26}O_4N_2$	382.1893	[12]
**tetracyclic monoterpene indole type, tMIA**				
32	corynantheidine	$C_{22}H_{28}O_3N_2$	368.2100	[7]
33	dihydrovallesiachotamine	$C_{21}H_{24}O_3N_2$	352.1787	[13]
34	isovallesiachotamine	$C_{21}H_{22}O_3N_2$	350.1630	[13]
35	hirsuteine	$C_{22}H_{26}O_3N_2$	366.1943	[14]
36	hirsutine	$C_{22}H_{28}O_3N_2$	368.2100	[14]
37	epi-allo-corynantheine	$C_{22}H_{26}O_3N_2$	366.1943	[1]
38	corynantheine	$C_{22}H_{26}O_3N_2$	366.1943	[15]
39	dihydrocorynantheine	$C_{22}H_{28}O_3N_2$	368.2100	[11]
40	geissoschizine methyl ether	$C_{22}H_{26}O_3N_2$	366.1943	[16]
41	4-geissoschizine *N*-oxide methyl ether	$C_{22}H_{26}O_4N_2$	382.1893	[17]
42	3-epi-geissoschizine methyl ether	$C_{22}H_{26}O_3N_2$	366.1943	[17]
43	gambireine	$C_{22}H_{26}O_4N_2$	382.1893	[18]
44	gambirine	$C_{22}H_{28}O_4N_2$	384.2049	[19]
45	isogambirine	$C_{22}H_{28}O_4N_2$	384.2049	[18]
46	glabratine	$C_{27}H_{34}O_9N_2$	530.2264	[3]
47	vallesiachotamine	$C_{21}H_{22}O_3N_2$	350.1630	[13]
48	villocarine A	$C_{22}H_{26}O_3N_2$	366.1943	[12]
49	hirsutine *N*-oxide	$C_{22}H_{28}O_4N_2$	384.2049	[20]
50	hirsuteine *N*-oxide	$C_{22}H_{26}O_4N_2$	382.1893	[17]
51	16,17-dehydro-*O*-demethylhirsuteine	$C_{21}H_{26}O_3N_2$	354.1943	[21]
**pentacyclic monoterpene oxindole type, pMOA**				
52	mitraphylline	$C_{21}H_{24}O_4N_2$	368.1736	[22]
53	isomitraphylline	$C_{21}H_{24}O_4N_2$	368.1736	[23]
54	mitraphyllic acid	$C_{20}H_{22}O_4N_2$	354.1580	[10]
55	isomitraphyllic acid	$C_{20}H_{22}O_4N_2$	354.1580	[24]
56	*O*-ethyl isomitraphylline	$C_{22}H_{26}O_4N_2$	382.1893	[23]
57	mitraphyllic acid-(16-1)-*β*-D-glucopyranoside	$C_{26}H_{32}O_9N_2$	516.2108	[24]
58	isomitraphyllic acid-(16-1)-*β*-D-glucopyranoside	$C_{26}H_{32}O_9N_2$	516.2108	[24]
59	pteropodic acid	$C_{20}H_{22}O_4N_2$	354.1580	[10]
60	isopteropodic acid	$C_{20}H_{22}O_4N_2$	354.1580	[10]
61	pterpodine	$C_{21}H_{24}O_4N_2$	368.1736	[25]
62	isopterpodine	$C_{21}H_{24}O_4N_2$	368.1736	[23]
63	rauniticine oxindole A	$C_{21}H_{24}O_4N_2$	368.1736	[26]
64	rauniticine oxindole B	$C_{21}H_{24}O_4N_2$	368.1736	[3]
65	speciophylline	$C_{21}H_{24}O_4N_2$	368.1736	[23]

66	uncarine A	$C_{21}H_{24}O_4N_2$	368.1736	[27]
67	uncarine F	$C_{21}H_{24}O_4N_2$	368.1736	[23]
68	uncaric acid A	$C_{20}H_{22}O_4N_2$	354.1580	[4]
69	uncarine B	$C_{21}H_{24}O_4N_2$	368.1736	[3]
70	rumberine	$C_{21}H_{24}O_5N_2$	384.1685	[28]
71	rotundifoline	$C_{22}H_{28}O_5N_2$	400.1998	[1]
72	isorotundifoline	$C_{22}H_{28}O_5N_2$	400.1998	[29]
73	gambirdine	$C_{21}H_{24}O_4N_2$	368.1736	[30]
74	isogambirdine	$C_{21}H_{24}O_4N_2$	368.1736	[30]
75	rauniticinic-allo acid B	$C_{20}H_{22}O_4N_2$	354.1580	[31]
76	rauniticine-allo oxindole B	$C_{21}H_{24}O_4N_2$	368.1736	[31]
77	mitraphylline *N*-oxide	$C_{21}H_{24}O_5N_2$	384.1685	[1]
78	isomitraphylline *N*-oxide	$C_{21}H_{24}O_5N_2$	384.1685	[1]
79	pterpodine *N*-oxide	$C_{21}H_{24}O_5N_2$	384.1685	[1]
80	isopterpodine *N*-oxide	$C_{21}H_{24}O_5N_2$	384.1685	[1]
81	speciophylline *N*-oxide	$C_{21}H_{24}O_5N_2$	384.1685	[1]
82	uncarine F *N*-oxide	$C_{21}H_{24}O_5N_2$	384.1685	[1]
83	uncarine B *N*-oxide	$C_{21}H_{24}O_5N_2$	384.1685	[32]
84	akuammigine pseudoindoxyl	$C_{21}H_{24}O_4N_2$	368.1736	[26]
85	rauniticine pseudoindoxyl	$C_{21}H_{24}O_4N_2$	368.1736	[26]
86	3-isorauniticine pseudoindoxyl	$C_{21}H_{24}O_4N_2$	368.1736	[26]
**pentacyclic monoterpene indole type, pMIA**				
87	ajmalicine	$C_{21}H_{24}O_3N_2$	352.1787	[26]
88	19-epi-ajmalicine	$C_{21}H_{24}O_3N_2$	352.1787	[26]
89	19-epi-3-iso-ajmalicine	$C_{21}H_{24}O_3N_2$	352.1787	[1]
90	3-epi-ajmalicine	$C_{21}H_{24}O_3N_2$	352.1787	[26]
91	akuammigine	$C_{21}H_{24}O_3N_2$	352.1787	[13]
92	rauniticine	$C_{21}H_{24}O_3N_2$	352.1787	[33]
93	14α-hydroxylrauniticine	$C_{21}H_{24}O_4N_2$	368.1736	[33]
94	14β-hydroxy-3-isorauniticine	$C_{21}H_{24}O_4N_2$	368.1736	[26]
95	3-isorauniticine	$C_{21}H_{24}O_3N_2$	352.1787	[26]
96	tetrahydroalstonine	$C_{21}H_{24}O_3N_2$	352.1787	[34]
97	diangoutengjian	$C_{21}H_{26}O_4N_2$	370.1893	[35]
98	tetrahydroalstonine *N*-oxide	$C_{21}H_{24}O_4N_2$	368.1736	[26]
99	rhynchophine	$C_{36}H_{38}O_{11}N_2$	674.2476	[13]
100	strictosidine lactam	$C_{26}H_{30}O_8N_2$	498.2002	[13]
101	vincoside lactam	$C_{26}H_{30}O_8N_2$	498.2002	[13]
102	2′-*O*-β-D-glucopyranosyl-11-hydroxyvincoside lactam	$C_{32}H_{40}O_{14}N_2$	676.2480	[11]
103	vincoside lactam A	$C_{26}H_{32}N_2O_{10}$	532.2057	[13]
**yohimbine type**				
104	pseudoyohimbine	$C_{21}H_{26}O_3N_2$	354.1943	[18]
105	alloyohimbine	$C_{21}H_{26}O_3N_2$	354.1943	[18]
106	3-epi-β-yohimbine	$C_{21}H_{26}O_3N_2$	354.1943	[18]
107	β-yohimbine	$C_{21}H_{26}O_3N_2$	354.1943	[18]
108	α-yohimbine	$C_{21}H_{26}O_3N_2$	354.1943	[18]
109	yohimbine	$C_{21}H_{26}O_3N_2$	354.1943	[18]
**pyridino-indolo-quinolizidinone type, angustine-type**				
110	angustine	$C_{20}H_{15}ON_3$	313.1215	[36]
111	angustidine	$C_{19}H_{15}ON_3$	301.1215	[36]
112	angustoline	$C_{20}H_{17}O_2N_3$	331.1321	[36]
**cadambine type**				
113	cadambine	$C_{27}H_{32}O_{10}N_2$	544.2057	[17]
114	3α-dihydrocadambine	$C_{27}H_{34}O_{10}N_2$	546.2214	[14]
115	3β-isodihydrocadambine	$C_{27}H_{34}O_{10}N_2$	546.2214	[17]
**3-Oxo-3,7-seco-monoterpene indoles**				

116	3-oxo-7-hydroxy-3,7-secorhy nchophylline	$C_{22}H_{28}O_6N_2$	416.1947	[37]
117	villocarine B	$C_{22}H_{26}O_6N_2$	414.1791	[12]
118	hirsutanine D	$C_{21}H_{24}O_6N_2$	401.1634	[32]
119	hirsutanine E	$C_{21}H_{24}O_6N_2$	401.1634	[32]
120	hirsutanine F	$C_{20}H_{24}O_5N_2$	372.1685	[32]
121	18α-hirsutanine F	$C_{20}H_{24}O_5N_2$	372.1685	[32]
**dimeric monoterpene indoles**				
122	callophylline A	$C_{43}H_{52}O_7N_4$	736.3836	[38]
123	callophylline B	$C_{43}H_{52}O_8N_4$	752.3785	[38]
124	callophylline	$C_{43}H_{52}O_7N_4$	736.3836	[18]
125	roxburghine A	$C_{31}H_{32}O_2N_4$	492.2525	[39]
126	roxburghine B	$C_{31}H_{32}O_2N_4$	492.2525	[39]
127	roxburghine C	$C_{31}H_{32}O_2N_4$	492.2525	[39]
128	roxburghine D	$C_{31}H_{32}O_2N_4$	492.2525	[39]
129	roxburghine E	$C_{31}H_{32}O_2N_4$	492.2525	[39]
130	roxburghine X	$C_{31}H_{32}O_2N_4$	492.2525	[40]
**others**				
131	uncariagambiriine	$C_{36}H_{32}O_8N_2$	620.2159	[41]
132	salacin	$C_{17}H_{20}O_3N_2$	300.1474	[37]
133	alkaloid us 7	$C_{22}H_{26}O_6N_2$	414.1791	[42]
134	alkaloid us 8	$C_{22}H_{26}O_6N_2$	414.1791	[42]
135	hirsutanine A	$C_{11}H_{15}O_3N$	209.1052	[43]
136	hirsutanine B	$C_{10}H_{13}O_3N$	195.0895	[43]
137	hirsutanine C	$C_{20}H_{24}O_5N_2$	372.1685	[43]
138	3-diethylamino-5-methoxy-1, 2-benzoquinone	$C_{11}H_{15}O_3N$	209.1052	[44]
139	3-ethylamino-5-methoxy-1, 2-benzoquinone	$C_9H_{10}O_3N$	181.0739	[44]
140	(-)-uncarilin A	$C_{38}H_{42}O_4N_6$	646.3268	[45]
141	(+)-uncarilin A	$C_{38}H_{42}O_4N_6$	646.3268	[45]
142	(-)-uncarilin B	$C_{38}H_{42}O_4N_6$	646.3268	[45]
143	(+)-uncarilin B	$C_{38}H_{42}O_4N_6$	646.3268	[45]

Table S2. Databases used and their detailed information

Database Name	Version	Access date	Website links	References
ClueGO	v2.5.4	2019-02-28	http://apps.cytoscape.org/apps/cluego	[46]
ConsensusPathDB	Release 34	2019-01-15	http://consensuspathdb.org/	[47]
Cytoscape	3.7.2	Latest Version 2019-05-13	https://cytoscape.org/	[48]
DrugBank	5.1.3	2019-04-02	https://www.drugbank.ca/	[49]
DrugBank	5.1.4	2019-07-02	https://www.drugbank.ca/	[49]
FAF-Drugs4	Version 4	2017-04-26	http://fafdrugs4.mti.univ-paris-diderot.fr/index.html	[50]
Metascape	Data updated	2019-08-14	http://metascape.org/gp/index.html#/main/step1	[51]

PharmMapper	monthly Version 2017	2016-01-01	http://www.lilab-ecust.cn/pharmmapper/	[52,53]
Similarity ensemble approach (SEA)	Latest Version	2019-03-26	http://sea.bkslab.org/	[54]
String	11.0	2019-01-19	https://string-db.org/	[55,56]
SwissTargetPrediction	Validation dataset	2019	http://www.swisstargetprediction.ch/index.php	[57]
TargetNet	1.0	2014-02-25	http://targetnet.scbdd.com/	[58]
Therapeutic Target Database (TTD)	Update 2018	2017-09-15	https://db.idrblab.org/ttd/	[59]
WebGestalt	WebGestalt 2019	2019-12-09	http://www.webgestalt.org/	[60]
Wikipathways	December 2019 Release	2019-12	https://www.wikipathways.org/index.php/WikiPathways	[61]

Table S3. *In vitro* BChE enzyme activity assay of galantamine as the positive control.

Conc. (μM)	Inhibition rate (%)
	galantamine
10	2.86
50	41.00
100	53.47
200	59.62
IC_{50}	18.21

References

1. Phillipson, J.D.; Hemingway, S.R.J.P. Alkaloids of Uncaria attenuata, U. orientalis and U. Canescens. **1975**, *14*, 1855-1863.
2. Kitajima, M.; Hashimoto, K.-i.; Yokoya, M.; Takayama, H.; Aimi, N.; SAKAI, S.-i.J.C.; bulletin, p. A New Gluco Indole Alkaloid, 3, 4-Dehydro-5-carboxystrictosidine, from Preruvian Una de Gato (Uncaria tomentosa). **2000**, *48*, 1410-1412.
3. Arbain, D.; Putri, M.; Sargent, M.; Syarif, M.J.A.J.o.C. The alkaloids of Uncaria glabrata. **1993**, *46*, 863-872.
4. Xin, W.B.; Chou, G.X.; Wang, Z.T.J.C.C.L. Two new alkaloids from the leaves of

Uncaria hirsuta Haviland. **2008**, *19*, 931-933.
5. Xin, W.-B.; Chou, G.-X.; Wang, Z.-T.J.P.l. Bis (monoterpenoid) indole alkaloid glucosides from Uncaria hirsuta. **2011**, *4*, 380-382.
6. Shimada, Y.; Goto, H.; Itoh, T.; SAKAKIBARA, I.; KUBO, M.; SASAKI, H.; TERASAWA, K.J.J.o.P.; Pharmacology. Evaluation of the protective effects of alkaloids isolated from the hooks and stems of Uncaria sinensis on glutamate-induced neuronal death in cultured cerebellar granule cells from rats. **1999**, *51*, 715-722.
7. Wang, K.; Zhou, X.-Y.; Wang, Y.-Y.; Li, M.-M.; Li, Y.-S.; Peng, L.-Y.; Cheng, X.; Li, Y.; Wang, Y.-P.; Zhao, Q.-S.J.J.o.n.p. Macrophyllionium and macrophyllines A and B, oxindole alkaloids from Uncaria macrophylla. **2011**, *74*, 12-15.
8. Phillipson, J.D.; Hemingway, S.R.J.P. Oxindole alkaloids from Uncaria macrophylla. **1973**, *12*, 2795-2798.
9. Yuan, D.; Ma, B.; Wu, C.; Yang, J.; Zhang, L.; Liu, S.; Wu, L.; Kano, Y.J.J.o.n.p. Alkaloids from the leaves of Uncaria rhynchophylla and their inhibitory activity on NO production in lipopolysaccharide-activated microglia. **2008**, *71*, 1271-1274.
10. Liu, H.-M.; Feng, X.-Z.J.P. Oxindole alkaloids from Uncaria sinensis. **1993**, *33*, 707-710.
11. Ma, B.; Wu, C.F.; Yang, J.Y.; Wang, R.; Kano, Y.; Yuan, D.J.H.C.A. Three new alkaloids from the leaves of Uncaria rhynchophylla. **2009**, *92*, 1575-1585.
12. Matsuo, H.; Okamoto, R.; Zaima, K.; Hirasawa, Y.; Ismail, I.S.; Lajis, N.H.; Morita, H.J.B.; chemistry, m. New vasorelaxant indole alkaloids, villocarines A–D from Uncaria villosa. **2011**, *19*, 4075-4079.
13. AIMI, N.; SHITO, T.; FUKUSHIMA, K.; ITAI, Y.; AOYAMA, C.; KUNISAWA, K.; SAKAI, S.; HAGINIWA, J.; YAMASAKI, K.J.C.; Bulletin, P. Studies on Plants containing Indole Alkaloids. VIII Indole Alkaloid Glycosides and Other Constituents of the Leaves of Uncaria rhynchophylla MIQ. **1982**, *30*, 4046-4051.
14. KOHDA, H.; NAMERA, A.; KOYAMA, A.; YAMASAKI, K.; TANI, T.J.C.; bulletin, p. Indole alkaloid production in callus cultures of Uncaria rhynchophylla (Miq.) Miquel. **1996**, *44*, 352-357.
15. Aimi, N.; Yamanaka, E.; Endo, J.; Sakai, S.-i.; Haginiwa, J.J.T.L. Conversion of oxindole alkaloids into indole alkaloids. **1972**, *13*, 1081-1084.
16. AIMI, N.; YAMANAKA, E.; SHINMA, N.; FUJIU, M.; KURITA, J.; SAKAI, S.; HAGINIWA, J.J.C.; Bulletin, P. Studies on Plants containing Indole Alkaloids. VI. Minor Bases of Uncaria rhynchophylla MIQ. **1977**, *25*, 2067-2071.
17. Qi, W.; Yue, S.-J.; Sun, J.-H.; Simpkins, J.W.; Zhang, L.; Yuan, D.J.J.o.A.n.p.r. Alkaloids from the hook-bearing branch of Uncaria rhynchophylla and their neuroprotective effects against glutamate-induced HT22 cell death. **2014**, *16*, 876-883.
18. Kam, T.-S.; Lee, K.-H.; Goh, S.-H.J.P. Alkaloid distribution in Malaysian uncaria. **1992**, *31*, 2031-2034.
19. Merlini, L.; Mondelli, R.; Nasini, G.; Hesse, M.J.T.l. Gambirine, a new indole alkaloid from uncaria gambier roxb. **1967**, *8*, 1571-1574.
20. Aimi, N.; Yamanaka, E.; Ogawa, M.; Kohmoto, T.; Mogi, K.; Sakai, S.J.H. Preparation

and stereochemistry of some indole alkaloid N-oxides. **1978**, *10*, 73-78.

21. Pan, H.; Yang, W.; Zhang, Y.; Yang, M.; Feng, R.; Wu, W.; Guo, D.J.A.; chemistry, b. An integrated strategy for the systematic characterization and discovery of new indole alkaloids from Uncaria rhynchophylla by UHPLC/DAD/LTQ-Orbitrap-MS. **2015**, *407*, 6057-6070.
22. Diyabalanage, T.K.; Kumarihamy, B.M.; Wannigama, G.P.; Jayasinghe, L.; Merlini, L.; Scaglioni, L.J.P. Alkaloids of Uncaria elliptica. **1997**, *45*, 1731-1732.
23. Monache, F.; Ferrari, F.; Marini-Bettolo, G.J.I.F.; scientifica, e. Alkaloids and procyanidins of an Uncaria sp. from Peru. **1976**, *31*, 527-535.
24. Liu, H.; Jiang, Z.; Feng, X.J.Y.x.x.b.A.p.S. New oxindole alkaloid glycosides from Uncaria sinensis. **1993**, *28*, 849-853.
25. Chan, K.J.P. The stereochemistry of pteropodine and isopteropodine. **1969**, *8*, 219-222.
26. Phillipson, J.D.; Supavita, N.J.P. Alkaloids of uncaria elliptica. **1983**, *22*, 1809-1813.
27. Wu, T.S.; Chan, Y.Y.J.J.o.t.C.C.S. Constituents of leaves of Uncaria hirsuta Haviland. **1994**, *41*, 209-212.
28. TANAHASHI, T.; TAKENAKA, Y.; KOBAYASHI, C.; WATSUJI, J.; NAGAKURA, N.; Cheng-Chang, C.J.N.m.生. Oxindole alkaloids from Uncaria setiloba. **1997**, *51*, 556.
29. Lala, P.J.F.-. Alkaloidal profile of Mitragyna stipulosa. **1985**.
30. Chan, K.J.T.l. Gambirdine and isogambirdine, the alkaloids from uncaria gambir (hunt) roxb. **1968**, *9*, 3403-3406.
31. Salim, F.; Ismail, N.H.; Awang, K.; Ahmad, R.J.M. Rauniticine-allo-oxindole B and rauniticinic-allo acid B, new heteroyohimbine-type oxindole alkaloids from the stems of Malaysian Uncaria longiflora var. pteropoda. **2011**, *16*, 6541-6548.
32. Pan, H.-q.; Yang, W.-z.; Zhao, D.; Luo, C.; Yao, C.-l.; Shi, X.-j.; Zhang, Y.-b.; Li, S.-y.; Bi, Y.; Wang, Z.J.F. New monoterpenoid oxindole alkaloid derivatives from the stems of Uncaria hirsuta Havil. and their cytotoxicity and tandem mass spectrometric fragmentation. **2017**, *116*, 85-92.
33. Arbain, D.; Ibrahim, S.; Sargent, M.V.; Skelton, B.W.; White, A.H.J.A.j.o.c. The alkaloids of Uncaria cf. glabrata. **1998**, *51*, 961-964.
34. Ponglux, D.; Supavita, T.; Verpoorte, R.; Phillipson, D.J.P. Alkaloids of Uncaria attenuata from Thailand. **1980**, *19*, 2013-2016.
35. Tao, Z.; Yi, Y.; Xu, Q.J.Y.x.x.b.A.p.S. Studies on the chemical constituents of Uncaria yunanensis Hsia. CC. **2001**, *36*, 120-122.
36. Phillipson, J.D.; Hemingway, S.R.; Bisset, N.G.; Houghton, P.J.; Shellard, E.J.J.P. Angustine and related alkaloids from species of Mitragyna, nauclea, uncaria, and Strychnos. **1974**, *13*, 973-978.
37. PONGLUX, D.; WONGSERIPIPATANA, S.; AIMI, N.; NISHIMURA, M.; ISHIKAWA, M.; SADA, H.; HAGINIWA, J.; SAKAI, S.-i.J.C.; bulletin, p. Structure and synthesis of two new types of oxindole alkaloids found from Uncaria salaccensis. **1990**, *38*, 573-575.
38. Kam, T.-S.; Lee, K.-H.; Goh, S.-H.J.P. Dimeric indole alkaloids from Uncaria callophylla. **1991**, *30*, 3441-3444.
39. Merlini, L.; Mondelli, R.; Nasini, G.; Hesse, M.J.T. The structure of roxburghines A–E,

new indole alkaloids from an Uncaria Sp. **1970**, *26*, 2259-2279.

40. Herath, W.; Sultanbawa, M.; Wannigama, G.; Cavé, A.J.P. Alkaloidal and other constituents of Uncaria elliptica and Canthium dicoccum. **1979**, *18*, 1385-1387.
41. Yoshikado, N.; Taniguchi, S.; Kasajima, N.; Ohashi, F.; Doi, K.I.; Shibata, T.; Yoshida, T.; Hatano, T.J.H. Uncariagambiriine and gambircatechol: Novel constituents of Uncaria gambir leaves. **2009**, *77*, 793-800.
42. Aimi, N.; Shimizu, T.; Sada, H.; Takayama, H.; Sakai, S.-i.; Wongseripipatana, S.; Ponglux, D.J.J.o.t.C.S., Perkin Transactions 1. Structures of Us-7 and Us-8: a new type of oxindole alkaloids isolated fromUncaria attenuata Korth. **1997**, 187-188.
43. Jia, J.-F.; Zhang, Y.; Huang, X.-J.; Zhang, S.-Y.; Tian, H.-Y.; Wang, L.; Ye, W.-C.J.N.p.r. New monoterpenoid alkaloids from the aerial parts of Uncaria hirsuta. **2014**, *28*, 971-975.
44. Qian, Z.; Lei, C.; Le-Jian, H.; Wen-Yuan, L.; Feng, F.; Wei, Q.J.C.j.o.n.m. Two new ortho benzoquinones from Uncaria rhynchophylla. **2016**, *14*, 232-235.
45. Geng, C.-A.; Huang, X.-Y.; Ma, Y.-B.; Hou, B.; Li, T.-Z.; Zhang, X.-M.; Chen, J.-J.J.J.o.n.p. (±)-Uncarilins A and B, dimeric isoechinulin-type alkaloids from Uncaria rhynchophylla. **2017**, *80*, 959-964.
46. Bindea, G.; Mlecnik, B.; Hackl, H.; Charoentong, P.; Tosolini, M.; Kirilovsky, A.; Fridman, W.H.; Pages, F.; Trajanoski, Z.; Galon, J. ClueGO: a Cytoscape plug-in to decipher functionally grouped gene ontology and pathway annotation networks. *Bioinformatics* **2009**, *25*, 1091-1093, doi:10.1093/bioinformatics/btp101.
47. Kamburov, A.; Stelzl, U.; Lehrach, H.; Herwig, R. The ConsensusPathDB interaction database: 2013 update. *Nucleic Acids Res* **2013**, *41*, D793-800, doi:10.1093/nar/gks1055.
48. Shannon, P.; Markiel, A.; Ozier, O.; Baliga, N.S.; Wang, J.T.; Ramage, D.; Amin, N.; Schwikowski, B.; Ideker, T. Cytoscape: a software environment for integrated models of biomolecular interaction networks. *Genome Res* **2003**, *13*, 2498-2504, doi:10.1101/gr.1239303.
49. Wishart, D.S.; Knox, C.; Guo, A.C.; Cheng, D.; Shrivastava, S.; Tzur, D.; Gautam, B.; Hassanali, M. DrugBank: a knowledgebase for drugs, drug actions and drug targets. *Nucleic Acids Res* **2008**, *36*, D901-906, doi:10.1093/nar/gkm958.
50. Lagorce, D.; Bouslama, L.; Becot, J.; Miteva, M.A.; Villoutreix, B.O. FAF-Drugs4: free ADME-tox filtering computations for chemical biology and early stages drug discovery. *Bioinformatics* **2017**, *33*, 3658-3660, doi:10.1093/bioinformatics/btx491.
51. Zhou, Y.; Zhou, B.; Pache, L.; Chang, M.; Khodabakhshi, A.H.; Tanaseichuk, O.; Benner, C.; Chanda, S.K. Metascape provides a biologist-oriented resource for the analysis of systems-level datasets. *Nat Commun* **2019**, *10*, 1523, doi:10.1038/s41467-019-09234-6.
52. Liu, X.; Ouyang, S.; Yu, B.; Liu, Y.; Huang, K.; Gong, J.; Zheng, S.; Li, Z.; Li, H.; Jiang, H. PharmMapper server: a web server for potential drug target identification using pharmacophore mapping approach. *Nucleic Acids Res* **2010**, *38*, W609-614, doi:10.1093/nar/gkq300.
53. Wang, X.; Shen, Y.; Wang, S.; Li, S.; Zhang, W.; Liu, X.; Lai, L.; Pei, J.; Li, H. PharmMapper 2017 update: a web server for potential drug target identification with a comprehensive target pharmacophore database. *Nucleic Acids Res* **2017**, *45*,

W356-W360, doi:10.1093/nar/gkx374.
54. Gfeller, D.; Michielin, O.; Zoete, V. Shaping the interaction landscape of bioactive molecules. *Bioinformatics* **2013**, *29*, 3073-3079, doi:10.1093/bioinformatics/btt540.
55. Szklarczyk, D.; Franceschini, A.; Wyder, S.; Forslund, K.; Heller, D.; Huerta-Cepas, J.; Simonovic, M.; Roth, A.; Santos, A.; Tsafou, K.P., et al. STRING v10: protein-protein interaction networks, integrated over the tree of life. *Nucleic Acids Res* **2015**, *43*, D447-452, doi:10.1093/nar/gku1003.
56. Szklarczyk, D.; Gable, A.L.; Lyon, D.; Junge, A.; Wyder, S.; Huerta-Cepas, J.; Simonovic, M.; Doncheva, N.T.; Morris, J.H.; Bork, P., et al. STRING v11: protein-protein association networks with increased coverage, supporting functional discovery in genome-wide experimental datasets. *Nucleic Acids Res* **2019**, *47*, D607-D613, doi:10.1093/nar/gky1131.
57. Daina, A.; Michielin, O.; Zoete, V. SwissTargetPrediction: updated data and new features for efficient prediction of protein targets of small molecules. *Nucleic Acids Res* **2019**, *47*, W357-W364, doi:10.1093/nar/gkz382.
58. Yao, Z.J.; Dong, J.; Che, Y.J.; Zhu, M.F.; Wen, M.; Wang, N.N.; Wang, S.; Lu, A.P.; Cao, D.S. TargetNet: a web service for predicting potential drug-target interaction profiling via multi-target SAR models. *J Comput Aided Mol Des* **2016**, *30*, 413-424, doi:10.1007/s10822-016-9915-2.
59. Chen, X.; Ji, Z.L.; Chen, Y.Z. TTD: Therapeutic Target Database. *Nucleic Acids Res* **2002**, *30*, 412-415, doi:10.1093/nar/30.1.412.
60. Liao, Y.; Wang, J.; Jaehnig, E.J.; Shi, Z.; Zhang, B. WebGestalt 2019: gene set analysis toolkit with revamped UIs and APIs. *Nucleic Acids Res* **2019**, *47*, W199-W205, doi:10.1093/nar/gkz401.
61. Slenter, D.N.; Kutmon, M.; Hanspers, K.; Riutta, A.; Windsor, J.; Nunes, N.; Melius, J.; Cirillo, E.; Coort, S.L.; Digles, D., et al. WikiPathways: a multifaceted pathway database bridging metabolomics to other omics research. *Nucleic Acids Res* **2018**, *46*, D661-D667, doi:10.1093/nar/gkx1064.
